# Risk factors for long-term cardiovascular post-acute sequelae of COVID-19 infection: A nested case-control study in Hong Kong

**DOI:** 10.1038/s44325-024-00011-z

**Published:** 2024-08-02

**Authors:** Qiuyan Yu, Min Fan, Celia Jiaxi Lin, David Tak Wai Lui, Kathryn Choon Beng Tan, Kai Hang Yiu, Ralph Kwame Akyea, Nadeem Qureshi, Francisco Tsz Tsun Lai, Eric Yuk Fai Wan, Xue Li, Esther Wai Yin Chan, Ian Chi Kei Wong, Celine Sze Ling Chui

**Affiliations:** 1 Centre for Safe Medication Practice and Research, Department of Pharmacology and Pharmacy, Li Ka Shing Faculty of Medicine, The University of Hong Kong, Hong Kong, China; 2 Laboratory of Data Discovery for Health (D24H), Hong Kong Science and Technology Park, Hong Kong, China; 3 School of Nursing, Li Ka Shing Faculty of Medicine, The University of Hong Kong, Hong Kong, China; 4 Department of Medicine, School of Clinical Medicine, Li Ka Shing Faculty of Medicine, The University of Hong Kong, Hong Kong, China; 5 Centre for Academic Primary Care, School of Medicine, University of Nottingham, Nottingham, United Kingdom; 6 Department of Family Medicine and Primary Care, School of Clinical Medicine, Li Ka Shing Faculty of Medicine, The University of Hong Kong, Hong Kong, China; 7 Department of Pharmacy, The University of Hong Kong-Shenzhen Hospital, Shenzhen, China; 8 The University of Hong Kong Shenzhen Institute of Research and Innovation, Shenzhen, China; 9 Research Department of Practice and Policy, School of Pharmacy, University College London, London, United Kingdom; 10 Aston Pharmacy School, Aston University, Aston Street, Birmingham, B4 7ET United Kingdom; 11 School of Public Health, Li Ka Shing Faculty of Medicine, The University of Hong Kong, Hong Kong, China

**Keywords:** Cardiovascular diseases, Endocrine system and metabolic diseases

## Abstract

People with COVID-19 can experience post-acute sequelae of SARS-CoV-2 (PASC). Studies on risk factors of PASC outcomes are ongoing, especially for endocrine system-related diseases that may impact the cardiovascular system. Cardiac-related PASC is one of the burdens after COVID-19 infection. This study aimed to examine the risk factors of cardiac-related PASC. In this nested case-control study, we obtained electronic health records (EHRs) database from the Hong Kong Hospital Authority. We defined cases as patients with at least one cardiac-related PASC and controls as patients without any cardiac-related PASC. We applied the incidence density sampling and matched controls to cases on age and sex at a 1:10 ratio. Multivariable conditional logistic regression was used to determine the associations between risk factors and cardiac-related PASC. A total of 455 individuals with cardiac-related PASC and matched 3,423 controls were obtained in the underlying cohort. COVID-19-associated hospitalisation (aOR: 1.41, 95% CI: 1.03–1.93) and peripheral vascular disease (aOR: 2.98, 95% CI: 1.31–6.79) were associated with an increased likelihood of cardiac-related PASC. Higher doses of the COVID-19 vaccine (2 doses: 0.68 [0.52–0.89]; ≥3 doses: 0.56 [0.40–0.78]) and more frequent healthcare utilization visits (aOR: 0.95, 95% CI: 0.92–0.97) were associated with a lower likelihood of cardiac-related PASC. This is the first study to examine risk factors of cardiac-related PASC among the Chinese population. We identified peripheral vascular disease and COVID-19-associated hospitalisation as the risk factors for cardiac-related PASC. COVID-19 vaccination was protective against cardiac-related PASC, which should be prioritized for high-risk patients.

## Introduction

SARS-CoV-2 infection (coronavirus disease 2019, COVID-19) has been reported to be associated with an increased risk of morbidity and mortality worldwide in the acute stage of infection within the first two weeks^1–3^. The Literature has demonstrated that people who have COVID-19 infection can experience some potential long-term symptoms and conditions following the acute stage, namely post-acute sequelae of SARS-CoV-2 (PASC)^4,5^. PASC can be defined as the persistence of symptoms or sequelae beyond three weeks of COVID-19 infection onset^6^. Increased risk of PASC involving multiple-organ systems, cardiovascular and all-cause mortality among patients with COVID-19 infection has been reported in recent studies^7,8^. Research studies have identified some risk factors associated with PASC outcomes, including the severity of symptoms during acute COVID-19 infection, increasing age, female sex, and pre-existing comorbidities^9,10^. Yet, studies are still ongoing regarding the risk factors of PASC, especially for endocrine system-related diseases which may have a significant impact on the cardiovascular system^11^. Various cardiovascular disease (CVD) including stroke, atrial fibrillation, and heart failure was reported as potential PASC within 12 months after COVID-19 infection^12,13^. The cardiac-related PASC was also reported among patients with COVID-19 infections but without hospitalisation during the acute stage^12,14^. Survivors of COVID-19 commonly experience cardiac-related PASC such as chest pain, heart palpitations, tachycardia, and fainting, with significant symptoms lasting for months after infection^15–17^.

Alpo Vuorio et al. have discussed the impact of the acute phase of COVID-19 on endothelial function leading to concern towards chronic metabolic conditions, especially in patients with familial hypercholesterolemia (FH) known as a metabolic inherited disease that will adversely affect endothelial function^18^. A follow-up study has highlighted the concerns related to the long-term effects of COVID-19 and the increased risk of complications and potentially poor outcomes among patients with FH^19^. A recent study from the US database also shows that COVID-19 increases the risk of myocardial infarction (MI) in FH patients with or without diagnosed atherosclerotic cardiovascular disease^20^. It was reported that FH was associated with an increased risk of long-term sustained cardiovascular risk following COVID-19^21^.

Considering the long-term effect following COVID-19 for patients with FH and the risk of FH itself developing CVD in an early stage, it is important to examine FH as a risk factor for cardiac-related PASC among patients with COVID-19 infection. Identifying the potential risk factors integrating FH status for cardiac-related PASC outcomes can provide information to help monitor and multidisciplinary care for COVID-19 survivors. In addition, lack of evidence presents the risk factors including the Sinovac-CoronaVac vaccination for cardiac-related PASC in the Chinese population. Given that risk factor profiles may vary across ethnicities, and Sinovac-CoronaVac was uniquely developed and used in China, it is necessary to include the vaccination to investigate the effect on cardiac-related PASC in the Chinese population. This study aims to examine the potential risk factors related to cardiac-related PASC among patients infected with COVID-19 infection.

## Results

**Fig. 1:**
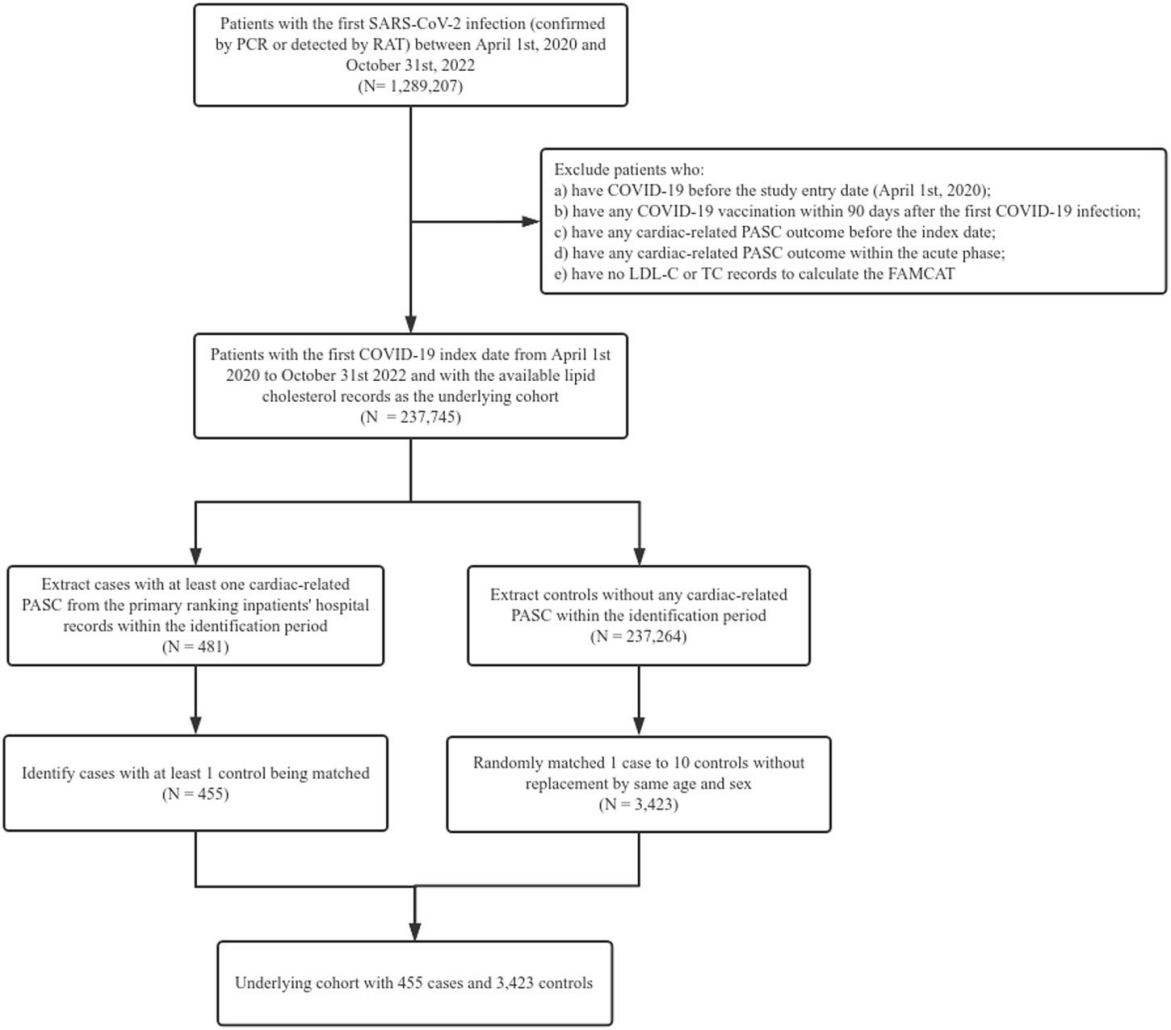
Flowchart of cohort selection. PCR polymerase chain reaction, RAT rapid antigen test, PASC post-acute sequelae of SARS-CoV-2, LDL-C low-density lipoprotein-cholesterol, TC total cholesterol.

We obtained 237,745 patients with COVID-19 infection who have at least one lipid cholesterol record (LDL-C or TC) as the underlying cohort, of whom 103,515 (43.5%) were men, with a mean age (±SD) of 58.8 (14.3) years. We identified 455 cases in the underlying cohort and matched 3423 controls at a 1:10 ratio. Figure 1 shows the underlying cohort selection process.

The baseline characteristics of the cases and controls are summarised in Table 1. The SMD of most baseline characteristics was ≤0.1 indicating that the variables were well-balanced between the cases and controls, except the age, Charlson Comorbidity Index, the doses of COVID-19 vaccines received, the antiviral prescription of COVID-19, the COVID-19 associated hospitalisation and healthcare utilisation, as well as the disease history such as peripheral vascular disease and chronic kidney disease. The most common comorbidities were hypertension (46.3%) and type 2 diabetes (22.2%). Cardiac-related PASC cases were older (mean age 70.4 vs. 68.7 years) and had a higher Charlson Comorbidity Index (0.6 vs. 0.5). More cardiac-related PASC cases did not receive COVID-19 vaccines (24.4% vs. 15.1%) while controls received more than 1 or 2 doses of COVID-19 vaccines (1 dose: 9.7% vs. 12.2%; 2 doses: 30.1% vs. 37.5%). Cardiac-related PASC cases were more prescribed with antiviral drugs for COVID-19 than controls (17.6% vs. 12.2%). In addition, more cardiac-related PASC cases have COVID-19-associated hospitalisation (16.9% vs. 8.3%) but less healthcare utilisation (mean visits 3.5 vs. 4.6).

**Table 1:** Baseline characteristics of the cases and controls

Baseline characteristic	Cases (*N* = 455)		Controls (*N* = 3423)		SMD^b^
	N/Mean	%/SD	N/Mean	%/SD	
**Demographics**					
Age, years^a^	70.4	13.4	68.7	12.0	0.134
Sex, male	273	60.0	2079	60.7	0.015
Charlson Comorbidity Index^a^	0.6	1.3	0.5	1.1	0.105
**Pre-existing morbidities**					
Peripheral vascular disease	9	2.0	19	0.6	0.128
Respiratory disease	21	4.6	130	3.8	0.041
Chronic obstructive pulmonary disease	21	4.6	130	3.8	0.041
Paralysis	0	0.0	0	0.0	<0.001
Type 2 diabetes	108	23.7	754	22.0	0.041
Chronic kidney disease	21	4.6	89	2.6	0.108
Mild liver disease	2	0.4	21	0.6	0.024
Moderate-severe liver disease	0	0.0	10	0.3	0.077
Ulcers	7	1.5	87	2.5	0.071
Rheumatoid arthritis and other Inflammatory polyarthropathies	5	1.1	32	0.9	0.016
Malignancy	29	6.4	195	5.7	0.028
Metastatic solid tumour	6	1.3	29	0.8	0.046
Hypertension	194	42.6	1600	46.7	0.083
Mental disorders	47	10.3	293	8.6	0.061
FH screened by FAMCAT^a^	0.0	0.0	0.0	0.0	0.020
**Doses of COVID-19 vaccines received**					0.258
0	111	24.4	518	15.1	
1	44	9.7	416	12.2	
2	137	30.1	1284	37.5	
3 or above	163	35.8	1205	35.2	
Antiviral Prescription for COVID-19^c^	80	17.6	417	12.2	0.152
COVID-19 associated hospitalisation	77	16.9	283	8.3	0.263
Healthcare utilisation within 2 years^a^	3.5	4.3	4.6	4.8	0.238

SMD Standardized mean difference.

^a^Age, Charlson Comorbidity Index, FH screened by FAMCAT, and healthcare utilisation are presented in mean ± Standard Deviation (SD).

^b^SMD ≤ 0.1 is considered a good balance between cases and controls.

^c^Antiviral prescription for COVID-19 was defined as prescribed Molnupiravir, Paxlovid, or Remdesivir within five days after being infected with COVID-19.

For the potential risk factors, we found that patients with COVID-19 associated hospitalisation (aOR: 1.41, 95% CI: 1.03–1.93) and peripheral vascular disease (aOR: 2.98, 95% CI: 1.31–6.79) had a greater likelihood of having cardiac-related PASC. Patients who received more doses of the COVID-19 vaccine were found to have a lower likelihood of having cardiac-related PASC (2 doses: 0.68 [0.52–0.89]; ≥3 doses: 0.56 [0.40–0.78]). Patients with more frequent healthcare utilisation within 2 years also had a lower risk of cardiac-related PASC (aOR: 0.95, 95% CI: 0.92–0.97). Table 2 shows the results of univariate and multivariable regression analyses with all the potential risk factors.

**Table 2:** Univariate regression and multivariable regression analysis results

	*N*^a^	Univariate regression analysis		Multivariable regression analysis	
Potential Risk Factors		OR (95% CI)	P-value	aOR (95% CI)	P-value
FH screened by FAMCAT	–	0.142 (0 - Inf)	0.878	0.50 (0.00 - Inf)	0.951
Vaccine status					
0–1 dose	1089	Ref		ref	
2 doses	1421	0.60 (0.47–0.79)	<0.001**	0.68 (0.52–0.89)	0.004*
≥3 doses	1368	0.49 (0.35–0.68)	<0.001***	0.56 (0.40–0.78)	<0.001**
Healthcare utilisation within 2 years	–	0.94 (0.92–0.97)	<0.001***	0.95 (0.92–0.97)	<0.001**
COVID-19 associated hospitalisation	360	1.66 (1.22–2.26)	0.001*	1.41 (1.03–1.93)	0.035*
Charlson Comorbidity Index	–	1.10 (1.02–1.19)	0.018*	1.04 (0.95–1.14)	0.365
Peripheral vascular disease	28	3.62 (1.62–8.10)	0.002*	2.98 (1.31–6.79)	0.009*
Hypertension	1794	0.83 (0.67–1.01)	0.062	1.01 (0.80–1.29)	0.913
Type 2 diabetes	862	1.13 (0.89–1.43)	0.323	1.16 (0.89–1.51)	0.275
Mental disorders	340	1.20 (0.86–1.68)	0.291	1.09 (0.77–1.54)	0.628

*OR* odds ratio, *aOR* adjusted odds ratio, 95% CI: 95% confidence interval.

*** *P*-value < 0.0001, ** *P*-value < 0.001, * *P*-value < 0.05.

^a^Number of observations: Continuous variables are not presenting an observation number.

### Subgroup and sensitivity analysis

Subgroup analyses conducted reported results in Supplementary Tables S3–S6. Patients with peripheral vascular disease have a greater likelihood of having cardiac-related PASC in the group aged 40-65 (aOR: 3.82, 95% CI: 1.02–14.31), men (aOR: 3.00, 95% CI: 1.20–7.50), Charlson Comorbidity Index below 4 (aOR: 2.64, 95% CI: 1.12–6.19), and received vaccine 3 doses or above (aOR: 9.79, 95% CI: 2.10–45.56). COVID-19-associated hospitalisation only presented as one of the risk factors in females (aOR: 1.73, 95%CI: 1.07–2.80) and the group of patients who received 2 doses of vaccine (aOR: 2.92, 95% CI: 1.23–6.91).

Sensitivity analyses reported relatively consistent results in the aforementioned risk factors (Supplementary Tables S7–S10). In the first sensitivity analysis with PCR-tested positive patients only, COVID-19-associated hospitalisation and peripheral vascular disease became an insignificant risk factor, while type 2 diabetes was significantly associated with the high risk of cardiac-related PASC (aOR: 1.40, 95%CI: 1.01–1.94). When we extend the case identification period to 22–180 days, COVID-19-associated hospitalisation also became an insignificant risk factor on the outcome, while peripheral vascular disease presented a much higher likelihood of having cardiac-related PASC (aOR: 4.50, 95% CI: 1.81–11.16). When we matched the cases and controls without limit and used the threshold of 0.0047 for FAMCAT to dichotomise FH status, the pattern of risk factors was similar to the primary analysis.

### Additional analysis

We found all of the COVID-19 vaccine subtypes were significantly associated with a lower likelihood of having cardiac-related PASC, except one dose of BNT162b2 and two doses BNT162b2 followed by one dose Sinovac-CoronaVac (Supplementary Table S11). We did not find a significant association between antiviral treatments and cardiac-related PASC (Supplementary Table S12).

### Post hoc analysis

We did not find any of the ICU admissions, COVID-19-associated pneumonia, or positivity of cardiac injury markers are significantly associated with cardiac-related PASC. The estimations of other risk factors are consistent with the main analysis (Supplementary Table S13).

## Discussion

This is the first population-based study examining cardiac-related PASC risk factors in the Chinese population. The study considered FH as one of the risk factors and estimated the effect of both Sinovac-CoronaVac and BNT162b2 COVID-19 vaccine in developing cardiac-related PASC among patients with COVID-19 infection. We found that COVID-19-associated hospitalisation and peripheral vascular disease were associated with an increased likelihood of cardiac-related PASC diagnosis in the database. COVID-19 vaccination and healthcare utilisation within 2 years were associated with a lower likelihood of cardiac-related PASC diagnosis. Our findings were fairly consistent in the sensitivity analyses using a variety of approaches to select cases, controls, match, and screen FH with a threshold.

Consistent with previous findings^22^, our study found that patients with COVID-19-associated hospitalization have a high risk of cardiac-related PASC. Currently, there is a lack of studies that specifically focus on cardiac-related PASC and investigating the Sinovac-CoronaVac and BNT162b2 COVID-19 vaccine safety and effectiveness related to thromboembolism and carditis outcomes. Our study indicates that having COVID-19 vaccination with increased doses, regardless of the subtypes, is protective against cardiac-related PASC, supporting the importance of being vaccinated and the benefit of vaccination outweighs risks^23,24^. In addition, the COVID-19 vaccine was found to be effective in reducing COVID-19-associated hospitalisation in our team research^25,26^, taking vaccination should be highly prioritised for those high-risk patients to protect against developing cardiac-related PASC. We further explored the impact of the severity associated with COVID-19 infection on cardiac-related PASC outcomes in the post hoc analysis. The findings showed that the added estimated factors are not significantly associated with cardiac-related PASC, with other factors remaining consistent. We believe this may indicate that COVID-19 hospitalisation is a strong indicator of severity for cardiac-related PASC.

Previous studies have shown that the presence of comorbidities is related to a significantly increased risk for general PASC development^9,27^. Our study added to the literature by examining comorbidities, including peripheral vascular disease, hypertension, type 2 diabetes, mental disorders, and FH as the potentially cardiac-related PASC risk factors. As the common risk factors of CVD outcomes, we only found peripheral vascular disease was significantly associated with a higher likelihood of cardiac-related PASC in this study. In the context of exclusion criteria in this study, we have excluded individuals with serious CVD that occurred before infection (e.g., MI, stroke, CAD, etc.). It is important to note that even individuals with milder CVD-related conditions may be at an exacerbated condition triggered by COVID-19 with an increased risk of developing cardiac-related PASC. Therefore, this group of patients should be well-managed with prioritised for vaccination to provide better protection, which was found to have a protective effect in our study.

In the subgroup analysis, we observed similar patterns of risk factor distribution. We found peripheral vascular disease was significantly associated with a higher likelihood of cardiac-related PASC in the male group instead of the female group, which may be due to the sample size that the percentage of men is larger than women. When we look into the baseline characteristics, it is interesting that patients with cardiac-related PASC are more likely to have antiviral prescriptions for COVID-19. This may indicate that patients who used antiviral drugs are likely to be sicker than those without antiviral drugs. In addition, patients with COVID-19 who are over 60 years old or have comorbidities like diabetes and CAD would be prescribed antiviral treatment. Since the prevalence of most comorbidities was higher among cardiac-related PASC cases than controls, this may also account for the higher usage of antiviral prescriptions. However, we did not find a significant association between antiviral drugs and cardiac-related PASC outcomes in the additional analysis.

Prior work has reported that pre-existing anxiety and depression are associated with an increased risk of PASC^28^. However, our study did not find a significant association between pre-existing mental disorders and the development of cardiac-related PASC. Another study conducted by Hill^10^ found that a pre-existing diagnosis of depression was associated with a higher risk of subsequent PASC, however, prior diagnoses of other mental health diagnoses (e.g., psychosis) were associated with lower risk. Different types of mental disorders may play different roles with different mechanisms in developing PASC. To further understand mental disorders on cardiac-related PASC, we may need to separate them into specific conditions to investigate the effect further.

While Alpo Vuorio et al. ^21^ reported that FH was associated with an increased risk of CVD after COVID-19, we did not find a significant association between FH and cardiac-related PASC. However, we believe this study demonstrated the application of FAMCAT in EHRs databases. Compared with the Dutch Lipid Clinic Network (DLCN), one of the internationally recommended clinical algorithms to detect FH^29^, screening for FH with FAMCAT in EHR is more practicable, which requires automated correction of LDL-C level with concurrent lipid-lowering medication parameters^30^. In contrast, DLCN is strongly determined by the LDL-C level without considering the effect of lipid-lowering medications. FAMCAT could enable passive surveillance to screen patients likely to have FH using EHRs.

In this nested case-control study, misclassification bias may be the common limitation. Within the case identification period, the controls in the nested cohort may have had any cardiac-related PASC outcome but have not yet been diagnosed in the database. It may lead to underestimation of certain conditions of cardiac-related PASC, including cardiomyopathy, atrial fibrillation, myocarditis, and pericarditis, which may not always present with obvious symptoms. To reduce the bias, we did a sensitivity analysis which prolonged the case identification period to allow more time for patients to get an accurate diagnosis, increasing the validity of case and control identification. In addition, the database used in this study has been validated with a high coding accuracy, especially for cardiovascular outcomes. Similar limitations were also presented on the patient inclusion with COVID-19 infection. This study includes those patients with COVID-19 tested PCR or reported RAT positive in the HK population. Yet, there is still a large proportion of HK residents who get the infection but have not officially tested or reported positive recorded in the database. We cannot capture this part of patients, though, we believe there is enough sample size in the whole set, with over 90% of HK residents already getting infections. Another limitation is that we only limited the data to those who have lipid measures due to the calculation of FH likelihood using FAMCAT. Nevertheless, our study is the first that evaluated the risk factors of cardiac-related PASC among the Chinese population, incorporating the effect of Sinovac-CoronaVac vaccine and FH screened by FAMCAT, the primary sample size enabling us to identify some statistically meaningful risk factors. Lastly, even if we found an association between peripheral vascular disease and cardiac-related PASC, the number of patients with peripheral vascular disease is small due to the incident cases. In addition, as a common limitation of many EHRs, smoking data is unavailable in our database. Disease symptoms, lifestyle, and socioeconomic status data may not be fully captured in the database. We used COVID-19-associated hospitalisation as a proxy for those unmeasured factors and disease severity in the analysis. Further studies with available data are required to validate the study findings. While the study findings were robust for the Hong Kong population, their generalizability to other countries or regions may be limited. Further studies on other populations may be needed to confirm the robustness of the results.

In conclusion, this study is the first to examine risk factors of cardiac-related PASC among the Chinese population. We identified some important risk factors such as peripheral vascular disease and COVID-19-associated hospitalisation. COVID-19 vaccination was protective against cardiac-related PASC, which should be prioritised for high-risk patients.

## Methods

### Data sources

In this nested case-control study, we retrieved the electronic health records (EHRs) from the Hong Kong Hospital Authority. The Hospital Authority is a statutory body that manages all public hospitals and their ambulatory clinics in Hong Kong, China^31^. The service is available to all Hong Kong residents (>7.3 million) covering approximately 80% of all routine hospital admissions and all patients with COVID-19 in Hong Kong^31^. The database has been used in previous studies involving COVID-19 vaccine safety surveillance and effectiveness^32–34^. The database has been validated with high coding accuracy and has been extensively used for conducting high-quality large population-based studies^35–37^. We obtained the death records from the Hong Kong Deaths Registry to identify mortality in this study. Information on vaccination status was provided by the Department of Health, The Government of Hong Kong Special Administrative Region who is in charge of the only mass COVID-19 vaccination programme in Hong Kong during the study period.

Anonymised longitudinal clinical healthcare data since 2016 was obtained for all individuals from the database. Relevant data included baseline demographic (sex, age and Charlson Comorbidity Index), pre-existing comorbidities captured by clinical diagnosis codes using International Classification of Diseases, Ninth Revision, Clinical Modification (ICD-9-CM) (Supplementary Table S1), COVID-19 vaccination status, antiviral prescription for COVID-19, COVID-19 associated hospitalisation and the healthcare utilisation at baseline.

### Study design and patient population

This is a nested case-control study conducted on patients aged 18 years or above. The underlying cohort was identified as patients with laboratory-confirmed SARS-CoV-2 infection (confirmed by positive polymerase chain reaction [PCR] test in throat swab, nasopharyngeal aspirate, or deep throat sputum specimens) or patients detected with SARS-CoV-2 virus proteins (antigens) in respiratory specimens by rapid antigen test [RAT] between April 1, 2020 to October 31, 2022 from the database.

We considered the earliest date of the laboratory-confirmed or RAT-detected positive diagnosis of COVID-19 infection as the index date for each patient in the underlying cohort. Patients with a history of COVID-19 before the study start date were excluded to ensure the inclusion of patients with first COVID-19 during the study period. Individuals who had any COVID-19 vaccination 90 days after the first COVID-19 infection were excluded.

We defined the case and control identification period from 22 days to 90 days after patients’ first COVID-19 infection (index date). The time between the index date and 21 days after was considered the acute phase. Individuals who had any cardiac-related PASC outcome before the index date and within the acute phase were also excluded. To ensure sufficient data to estimate FH likelihood, patients with at least one low-density lipoprotein-cholesterol (LDL-C) or total cholesterol (TC) record before the index date were included as the underlying cohort. Eligible individuals were followed up from the index date until each cardiac-related PASC outcome event occurred, death, or study end date (January 31, 2023), whichever occurred earlier.

### Cases and controls

We identified the cases as patients who have at least one cardiac-related PASC in the identification period. The definition of cardiac-related PASC outcomes of this study was selected based on previous evidence on the risk of PASC, which includes incidences of stroke, MI, heart failure, atrial fibrillation, coronary artery disease (CAD), myocarditis and pericarditis, deep vein thrombosis, cardiomyopathy, and cardiovascular mortality^12,38–41^. The cardiac-related PASC diagnoses were identified from the primary ranking in-patients’ hospital records based on the ICD-9-CM code and death records were identified by the ICD-10-CM code in the database (Supplementary Table S2). We identified the controls as patients without any cardiac-related PASC in the identification period. The incidence density sampling was applied to select controls to obtain unbiased results^42^, in which the cases were allowed to be controls before their incident cardiac-related PASC. The pool of eligible controls includes all cohort members, with the exception of the index case itself. Up to ten controls were randomly selected without replacement from this pool of eligible controls to form the incidence density sampled risk set for the index case. Controls were matched to cases on attained age and sex. One control can be matched to more than one case.

### Risk factors

We defined the potential exposures of risk factors as demographic (age, sex, and Charlson Comorbidity Index), vaccination status (0-1 doses, 2 doses, ≥3 doses), COVID-19 associated hospitalisation, outpatient healthcare utilisation visits, and a list of clinical diagnosis history (peripheral vascular disease, hypertension, type 2 diabetes, mental disorders, FH), reference from existing literature^38^ and clinical expertise. As it is difficult to identify individuals with FH using the ICD-9-CM code in the EHR database, we used a case ascertainment tool named FAMCAT to measure the FH likelihood (Supplementary Information). FAMCAT is one of the validated tools in screening FH using the EHR database, enabling clinicians to estimate the probability of having FH^43^. We used the information before the index date to identify patients’ age, sex, Charlson Comorbidity Index, and FAMCAT probability. The vaccination status was defined as the doses of vaccination received at least 14 days before the index date. We defined COVID-19 associated hospitalisation as hospitalisation within 14 days after the first COVID-19 infection and healthcare utilisation as the count of outpatient visits within two years before the index date. The disease history was defined as either inpatient or outpatient diagnosis before the index date.

### Statistical analysis

Descriptive statistics were used to report the characteristics of cases and controls at baseline. We estimated the standardised mean difference (SMD) between the cases and controls, with SMD ≤ 0.1 regarded as a sufficient balance between case and control groups. Univariate analysis was conducted using conditional logistic regression to estimate the odds ratio (OR) and 95% confidence interval (95% CI) of each potential risk factor on the cardiac-related PASC outcomes among individuals with COVID-19 infection in the underlying cohort. We further conducted the multivariable conditional logistic regression for all the risk factors estimated in the univariate analysis presenting the adjusted odds ratio (aOR).

### Subgroup and sensitivity analysis

We conducted the subgroup analyses with patients stratified by 1) age (<40, ≤65, >65); 2) sex; 3) Charlson Comorbidity Index (<4, ≥4); and 4) COVID-19 vaccination status prior to the index date (0–1 dose, 2 doses, ≥3 doses). We performed the sensitivity analyses by 1) defining the underlying cohort as patients with laboratory-confirmed SARS-CoV-2 infection (confirmed by PCR) only; 2) defining the cases as patients who have any cardiac-related PASC outcomes from 22 days to 180 days after the first tested positive date of COVID-19 infection; 3) matching cases and controls as much as possible without 1:10 limit; and 4) applying FAMCAT with a threshold of 0.0047 to dichotomise FH status.

### Additional analysis

We performed an additional analysis by investigating the COVID-19 vaccination subtypes on the cardiac-related PASC. Vaccine subtypes were classified as one dose Sinovac-CoronaVac, one dose BNT162b2, two doses Sinovac-CoronaVac, two doses BNT162b2, three doses Sinovac-CoronaVac, three doses BNT162b2, two doses Sinovac-CoronaVac followed by one dose BNT162b2, two doses BNT162b2 followed by one dose Sinovac-CoronaVac. Patients who received four doses or above were categorised as three doses depending on their first three dose subtypes. We further conducted an additional analysis by including antiviral treatments as a potential risk factor for cardiac-related PASC. Antiviral treatments for COVID-19 were defined as prescribed Molnupiravir, Paxlovid, or Remdesivir within five days after being infected with COVID-19.

### Post hoc analysis

To explore the impact of severity of COVID-19 on cardiac-related PASC, we conducted a post hoc analysis by including ICU admission, COVID-19-associated pneumonia, and positivity of cardiac injury markers in the model. We identified patients who presented any diagnosis with an ICD9 code of 519.8 as having COVID-19-associated pneumonia. We identified patients with detected positive myoglobin or higher test results of creatine kinase or troponin than their reference upper level recorded in the database as having a cardiac injury. All ICU admissions, COVID-19-associated pneumonia, and positivity of cardiac injury markers were identified within 14 days after the index date to reflect the severity of COVID-19 infection.

All statistical analyses were conducted using R version 4.1.2 (R Foundation for Statistical Computing, Vienna, Austria). All significance tests were two-tailed and 95% CI excluding 1.0 was taken to indicate statistical significance. Two investigators (QY and MF) conducted each statistical analysis independently for quality assurance.

Ethical approval for this study was granted by the Institutional Review Board of the University of HK/HA HK West Cluster (UW20-556 and UW21-149) and Department of Health, HK (LM21/2021 and LM175/2022) with an exemption for informed consent from participants as patients’ confidentiality was maintained in this nested case-control study.

## Supplementary information

Supplemental Appendix

STROBE_checklist_case-control

## Data Availability

The data underlying this article are available from the Hong Kong Hospital Authority and cannot be shared publicly as stipulated in the Institutional Review Board application protocol. Inquiries for data access can be made at enquiry@ha.org.hk.
