# Supplementary material for: Risk factors for long-term cardiovascular post-acute sequelae of COVID-19 infection: A nested case-control study in Hong Kong
Source: NPJ Cardiovasc Health. 2024 Aug 2;1:10. doi: 10.1038/s44325-024-00011-z (PMC12912400; doi:10.1038/s44325-024-00011-z)
Supplement: Supplementary file 1 — Supplemental Appendix [file 44325_2024_11_MOESM1_ESM.pdf]

Supplemental Appendix

Table of Contents

1. Supplementary Information 2

2. Supplementary Tables 4

2.1 Supplementary Table S1. Diagnosis codes for all baseline disease conditions 4

2.2 Supplementary Table S2. List of cardiac-related post-acute sequelae of COVID-19 (PASC) ... 5

2.3 Supplementary Table S3. Subgroup analyses stratified by age 6

2.4 Supplementary Table S4. Subgroup analyses stratified by sex 8

2.5 Supplementary Table S5. Subgroup analyses stratified by Charlson Comorbidity Index 10

2.6 Supplementary Table S6. Subgroup analyses stratified by vaccine status 11

2.7 Supplementary Table S7. Sensitivity analyses with PCR tested positive patients only 13

2.8 Supplementary Table S8. Sensitivity analyses with cases identification period extended to 22-180 days 14

2.9 Supplementary Table S9. Sensitivity analyses with matching process without 1:10 limit 15

2.10 Supplementary Table S10. Sensitivity analyses with a threshold of 0.0047 for FAMCAT to screen FH 16

2.11 Supplementary Table S11. Additional analysis stratified by vaccine subtypes 17

2.12 Supplementary Table S12. Additional analysis with antiviral treatments included 18

2.13 Supplementary Table S13. Post hoc analysis with severity indicators included 19

3. Supplementary References 20

1. Supplementary Information

Introduction of FAMCAT

This study examined a list of risk factors including familial hypercholesterolemia (FH) for cardiac-related PASC. As mentioned in the Methods of the manuscript, the pre-existing comorbidities (disease history) were captured by the ICD-9-CM code using either inpatient or outpatient diagnosis information. However, we did not use the ICD-9-CM code to capture the FH diagnosis because it is not available for coding FH in the electronic health records (EHRs) database.

While FH is still underdiagnosed in the population, clinical diagnosis with validated diagnosis criteria or tools can help screen high-risk patients likely to have FH in general practice.[1] Using the EHR database, Familial Hypercholesterolaemia Case Ascertainment Tool (FAMCAT) is one of the tools confirmed as a good clinical tool in screening FH, enabling clinicians to estimate the probability of having FH.[2] Parameters such as cholesterol level, lipid-lowering medications and CVD-related family history are accounted for in the FAMCAT algorithm.[3]

Application and Interpretation of FAMCAT

In the primary analysis, we applied FAMCAT to measure the FH likelihood as a continuous variable in the regression model. In the sensitivity analysis, we applied FAMCAT with a threshold of 0.0047[4] to dichotomise FH status into a categorical variable in the regression model. It is noted that family history information is not available in our database. Therefore, we may obtain an underestimated probability of having FH for each eligible patient. Nevertheless, the performance of the algorithm without family history variables was still high with an AUC of 0.82.[5] We treated all missing family history records as none in the underlying cohort.

Discussion of using FAMCAT

The study findings add to the growing body of evidence that integrates the FAMCAT algorithm into measuring FH as a risk factor. This study also demonstrated the application of FAMCAT in our EHR database. With the automated correction of LDL-C level with concurrent lipid-lowering medication, screening for FH using the FAMCAT algorithm is more practicable in EHRs. FAMCAT could enable passive surveillance using EHRs, which could help inform the in-time therapy to optimise the primary care and prevention treatment for future CVD risk among those patients who are likely to have FH, with an expectation of stabilising their level of lipid serum continuously.

In this study, we did not find a significant association between FH screened by FAMCAT and cardiac-related PASC. We also obtained an underestimated probability of having FH, leading to fewer patients being screened as FH due to the lack of family history in the database. Nevertheless, we treated all missing family history records as none in the underlying cohort. In this case, there is no differential missingness of the family history across the case and control groups. Our estimation of the FAMCAT probability would be a conservative estimation of the FH likelihood.

For further applying the FAMCAT algorithm as an automatic screening tool in Hong Kong, validation and recalibration of the algorithm and adjustment of the threshold fit in Hong Kong data are required to increase the discrimination accuracy of the FH patients screening.

Difference of FH between cases and controls

Since we conducted the sensitivity analysis by using the FAMCAT with a threshold to dichotomise into FH status as a categorical variable, we want to check whether there is a difference in FH between the two groups. Therefore, we applied McNemar's Chi-squared test with continuity correction to test the differences in FH status screened by FAMCAT between the cases and controls. The results indicated a statistically significant difference in FH status screened by FAMCAT with the threshold set at 0.0047 between the cases and controls (P-value < 0.05).

2. Supplementary Tables

2.1 Supplementary Table S1. Diagnosis codes for all baseline disease conditions

**Pre-existing morbidities**	**ICD-9-CM code**
Peripheral vascular disease	441, 443.9, 785.4
Respiratory disease	416.8, 416.9, 490-496, 500-505, 506.4, 508.1, 508.8
Chronic obstructive pulmonary disease	490-496, 500-505, 506.4
Paralysis	342, 344.1
Type 2 diabetes	250.00, 250.02, 250.10, 250.12, 250.20, 250.22, 250.30, 250.32, 250.40, 250.42, 250.50, 250.52, 250.60, 250.62, 250.70, 250.72, 250.80, 250.82, 250.90, 250.92
Chronic kidney disease	582, 585, 586, 588, 583.0-583.2, 583.4, 583.6, 583.7
Mild liver disease	571.2, 571.4-571.6
Moderate-severe liver disease	456.0-456.2, 572.2-572.4. 572.8
Ulcers	531-534
Rheumatoid arthritis and other Inflammatory polyarthropathies	710.0, 710.1, 710.4, 714.0, 714.1, 714.2, 714.81, 725
Malignancy	140-149, 150-159, 180-189, 170-172, 174, 175, 176, 179, 160-165, 190-195, 200-208
Metastatic solid tumour	196-199
Hypertension	401-405, 437.2
Mental disorders	290-299, 300-319

2.2 Supplementary Table S2. List of cardiac-related post-acute sequelae of COVID-19 (PASC)

**Cardiac-related PASC outcomes**	**ICD-9-CM code**
Myocardial infarction	410
Heart failure	428,398.91,402.01,402.11,402.91,404.01,404.03, 404.11,404.13,404.91,404.93
Stroke	430-438
Atrial fibrillation	427.3
Coronary artery disease	410-414, 36.0, 36.1
Deep vein thrombosis	453
Myocarditis and pericarditis	422, 429.0, 420.9, 423.9
Cardiomyopathy	425.4, 425.5, 425.7, 425.8, 425.9
Cardiovascular mortality (ICD-10-CM)	I09.81, I21.x, I22.x, I24.x, I25.2, I42.5, I42.6, I42.8, I43, I42.7, I48.x, I50.x, I65- I69

2.3 Supplementary Table S3. Subgroup analyses stratified by age

Age < 40

Potential Risk Factors	N[a]	Univariate regression analysis		Multivariable regression analysis	
		OR (95% CI)	P-value	aOR (95% CI)	P-value
FH screened by FAMCAT	-	0.00 (0.00 – Inf)	0.389	0.00 (0.00 – Inf)	0.123
Vaccine status					
0-1 dose	15	ref		ref	
2 doses	31	0.11 (0.01 - 1.05)	0.055	12.46 (0.00 - Inf)	0.548
≥3 doses	10	0.00 (0.00 – Inf)	0.999	0.00 (000 - Inf)	0.999
Healthcare utilisation within 2 years	-	0.95 (0.78 - 1.15)	0.579	0.41 (0.04 - 3.90)	0.439
COVID-19 associated hospitalisation	4	17.59 (1.65 - 187.69)	0.017	Inf (0.00 - Inf)	0.177
Charlson Comorbidity Index	-	1.96 (0.49 - 7.86)	0.345	0.00 (0.00 - Inf)	0.999
Peripheral vascular disease	0	-	-	-	-
Hypertension	16	1.63 (0.25 - 10.77)	0.613	Inf (0.00 - Inf)	0.147
Type 2 diabetes	7	5.57 (1.01 - 30.75)	0.049	Inf (0.00 - Inf)	0.999
Mental disorders	13	0.60 (0.06 - 6.01)	0.665	0.07 (0.00 - Inf)	0.814

Age > 40 & <= 65

Potential Risk Factors	N[a]	Univariate regression analysis		Multivariable regression analysis	
		OR (95% CI)	P-value	aOR (95% CI)	P-value
FH screened by FAMCAT	-	20.32 (0.00 - Inf)	0.761	21.35 (0 - Inf)	0.757
Vaccine status					
0-1 dose	232	ref		ref	
2 doses	650	0.58 (0.36 - 0.92)	0.020*	0.64 (0.40 - 1.04)	0.069
≥3 doses	597	0.50 (0.29 - 0.87)	0.013	0.59 (0.33 - 1.04)	0.067
Healthcare utilisation within 2 years	-	0.95 (0.91 - 0.99)	0.020*	0.95 (0.90 – 1.00)	0.035*
COVID-19 associated hospitalisation	52	2.25 (1.10 - 4.60)	0.026	1.77 (0.83 - 3.77)	0.143
Charlson Comorbidity Index	-	1.21 (1.05 - 1.39)	0.008	1.12 (0.96 - 1.32)	0.156
Peripheral vascular disease	10	5.85 (1.64 - 20.89)	0.007	3.82 (1.02 - 14.31)	0.047*
Hypertension	625	0.95 (0.68 - 1.33)	0.761	1.11 (0.75 - 1.64)	0.596
Type 2 diabetes	268	1.07 (0.69 - 1.64)	0.768	0.97 (0.61 - 1.56)	0.906
Mental disorders	116	0.94 (0.49 - 1.79)	0.841	0.90 (0.46 - 1.76)	0.754

**Age > 65**					
**Potential Risk Factors**	**N[a]**	**Univariate regression analysis**		**Multivariable regression analysis**	
		**OR (95% CI)**	**P-value**	**aOR (95% CI)**	**P-value**
FH screened by FAMCAT	-	0.00 (0.00 – Inf)	0.370	0.00 (0.00 - Inf)	0.470
Vaccine status					
0-1 dose	811	ref		ref	
2 doses	812	0.57 (0.41 - 0.79)	<0.001**	0.62 (0.45 - 0.87)	0.005*
≥3 doses	720	0.52 (0.34 - 0.80)	0.003*	0.60 (0.39 - 0.92)	0.018*
Healthcare utilisation within 2 years	-	0.94 (0.91 – 0.97)	<0.001***	0.94 (0.90 - 0.98)	0.002*
COVID-19 associated hospitalisation	303	1.48 (1.05 - 2.08)	0.025*	1.27 (0.89 - 1.81)	0.193
Charlson Comorbidity Index	-	1.07 (0.97 - 1.18)	0.166	1.03 (0.92 - 1.15)	0.588
Peripheral vascular disease	23	2.10 (0.76 - 5.78)	0.151	1.863 (0.658 - 5.271)	0.241
Hypertension	1182	0.72 (0.56 - 0.93)	0.012*	0.889 (0.646 - 1.223)	0.469
Type 2 diabetes	578	1.13 (0.85 - 1.51)	0.398	1.208 (0.868 - 1.682)	0.263
Mental disorders	210	1.37 (0.92 - 2.05)	0.122	1.259 (0.83 - 1.911)	0.278

Note: OR: odds ratio, aOR: adjusted odds ratio, 95% CI: 95% confidence interval, results that filled in blank may be due to the small sample size

*** P-value < 0.0001, ** P-value < 0.001, * P-value < 0.05

a Number of observations: Continuous variables are not presenting an observation number

2.4 Supplementary Table S4. Subgroup analyses stratified by sex

Men

Potential Risk Factors	N[a]	Univariate regression analysis		Multivariable regression analysis	
		OR (95% CI)	P-value	aOR (95% CI)	P-value
FH screened by FAMCAT	-	6.88 (0.00 – Inf)	0.931	3.99 (0 - Inf)	0.948
Vaccine status					
0-1 dose	595	ref		ref	
2 doses	893	0.66 (0.47 - 0.94)	0.019	0.70 (0.49 - 0.99)	0.043*
≥3 doses	864	0.61 (0.40 - 0.93)	0.021	0.66 (0.43 - 1.01)	0.053
Healthcare utilisation within 2 years	-	0.95 (0.92 - 0.98)	0.001*	0.94 (0.90 - 0.98)	0.001*
COVID-19 associated hospitalisation	225	1.34 (0.89 - 2.02)	0.156	1.18 (0.78 - 1.80)	0.436
Charlson Comorbidity Index	-	1.07 (0.97 - 1.18)	0.188	1.04 (0.92 - 1.16)	0.548
Peripheral vascular disease	25	3.12 (1.28 - 7.64)	0.013	3.00 (1.20 - 7.50)	0.019*
Hypertension	1065	0.94 (0.73 - 1.22)	0.644	1.21 (0.89 - 1.65)	0.227
Type 2 diabetes	572	1.02 (0.75 - 1.37)	0.923	1.03 (0.73 - 1.45)	0.863
Mental disorders	188	1.04 (0.65 - 1.66)	0.874	0.95 (0.58 - 1.54)	0.831

Women

Potential Risk Factors	N[a]	Univariate regression analysis		Multivariable regression analysis	
		OR (95% CI)	P-value	aOR (95% CI)	P-value
FH screened by FAMCAT	-	0.43 (0.00 – Inf)	0.952	0.88 (0 - Inf)	0.992
Vaccine status					
0-1 dose	478	ref		ref	
2 doses	548	0.48 (0.31 - 0.73)	<0.001**	0.55 (0.36 - 0.84)	0.005*
≥3 doses	500	0.33 (0.19 - 0.56)	<0.001***	0.38 (0.22 - 0.66)	<0.001**
Healthcare utilisation within 2 years	-	0.94 (0.90 - 0.98)	0.003*	0.96 (0.91 - 1.00)	0.073
COVID-19 associated hospitalisation	140	2.14 (1.34 - 3.41)	0.001*	1.73 (1.07 - 2.80)	0.027*
Charlson Comorbidity Index	-	1.17 (1.02 - 1.35)	0.029*	1.14 (0.97 - 1.34)	0.104
Peripheral vascular disease	8	2.62 (0.52 - 13.19)	0.244	1.60 (0.30 - 8.64)	0.585
Hypertension	718	0.69 (0.50 - 0.96)	0.026*	0.76 (0.51 - 1.14)	0.183
Type 2 diabetes	317	1.21 (0.83 - 1.76)	0.325	1.19 (0.78 - 1.82)	0.427

Mental disorders	146	1.47 (0.90 - 2.38)	0.121	1.24 (0.75 - 2.07)	0.398

Note: OR: odds ratio, aOR: adjusted odds ratio, 95% CI: 95% confidence interval

*** P-value < 0.0001, ** P-value < 0.001, * P-value < 0.05

a Number of observations: Continuous variables are not presenting an observation number

2.5 Supplementary Table S5. Subgroup analyses stratified by Charlson Comorbidity Index

CCI < 4

**Potential Risk Factors**	**N**[a]	**Univariate regression analysis**		**Multivariable regression analysis**	
		**OR (95% CI)**	**P-value**	**aOR (95% CI)**	**P-value**
FH screened by FAMCAT	-	3475.60 (0.00 – Inf)	0.671	Inf (0.00 - Inf)	0.523
Vaccine status					
0-1 dose	1003	ref		ref	
2 doses	1402	0.61 (0.46 - 0.79)	<0.001**	0.66 (0.50 - 0.87)	0.003*
≥3 doses	1323	0.51 (0.36 - 0.71)	<0.001**	0.56 (0.40 - 0.79)	<0.001**
Healthcare utilisation within 2 years	-	0.95 (0.93 - 0.98)	<0.001***	0.95 (0.92 - 0.98)	0.001*
COVID-19 associated hospitalisation	333	1.58 (1.15 - 2.16)	0.005*	1.35 (0.98 - 1.88)	0.071
Charlson Comorbidity Index	-	1.11 (0.98 - 1.27)	0.107	1.03 (0.88 - 1.21)	0.691
Peripheral vascular disease	29	2.98 (1.31 - 6.80)	0.009*	2.64 (1.12 - 6.19)	0.026*
Hypertension	1705	0.81 (0.66 - 1.00)	0.050	0.98 (0.76 - 1.26)	0.870
Type 2 diabetes	788	1.13 (0.88 - 1.44)	0.336	1.19 (0.89 - 1.60)	0.241
Mental disorders	314	1.32 (0.94 - 1.85)	0.114	1.18 (0.83 - 1.68)	0.365

Note: The results of the subgroup patients with CCI>=4 were not presented due to the small sample size (2 cases); OR: odds ratio, aOR: adjusted odds ratio, 95% CI: 95% confidence interval

*** P-value < 0.0001, ** P-value < 0.001, * P-value < 0.05

a Number of observations: Continuous variables are not presenting an observation number

2.6 Supplementary Table S6. Subgroup analyses stratified by vaccine status

Vaccine 0-1 dose

Potential Risk Factors	N[a]	Univariate regression analysis		Multivariable regression analysis	
		OR (95% CI)	P-value	aOR (95% CI)	P-value
FH screened by FAMCAT	-	Inf (0.00 – Inf)	0.725	Inf (0.00 – Inf)	0.545
Vaccine status					
0-1 dose	1063				
2 doses	0				
≥3 doses	0				
Healthcare utilisation within 2 years	-	0.97 (0.93 - 1.01)	0.150	0.95 (0.90 - 1.00)	0.055
COVID-19 associated hospitalisation	169	1.05 (0.65 - 1.70)	0.839	0.94 (0.56 - 1.56)	0.800
Charlson Comorbidity Index	-	1.06 (0.94 - 1.20)	0.318	1.03 (0.89 - 1.18)	0.706
Peripheral vascular disease	12	2.48 (0.65 - 9.52)	0.185	2.05 (0.52 - 8.09)	0.307
Hypertension	485	1.09 (0.75 - 1.58)	0.661	1.26 (0.80 - 1.98)	0.314
Type 2 diabetes	277	1.29 (0.87 - 1.92)	0.212	1.33 (0.85 - 2.08)	0.209
Mental disorders	137	0.92 (0.54 - 1.58)	0.770	0.93 (0.53 - 1.61)	0.783

Vaccine 2 dose

Potential Risk Factors	N[a]	Univariate regression analysis		Multivariable regression analysis	
		OR (95% CI)	P-value	aOR (95% CI)	P-value
FH screened by FAMCAT	-	Inf (0.00 – Inf)	0.700	Inf (0.00 - Inf)	0.553
Vaccine status					
0-1 dose	0				
2 doses	1024				
≥3 doses	0				
Healthcare utilisation within 2 years	-	0.94 (0.90 - 0.99)	0.014	0.92 (0.87 - 0.98)	0.008*
COVID-19 associated hospitalisation	33	2.24 (0.98 - 5.14)	0.057	2.92 (1.23 - 6.91)	0.015*
Charlson Comorbidity Index	-	1.10 (0.92 - 1.31)	0.312	1.09 (0.90 - 1.32)	0.370
Peripheral vascular disease	4	3.75 (0.33 - 43.07)	0.289	5.53 (0.43 - 70.74)	0.189
Hypertension	483	0.82 (0.56 - 1.21)	0.311	1.09 (0.68 - 1.75)	0.719
Type 2 diabetes	220	0.98 (0.60 - 1.59)	0.924	1.07 (0.62 - 1.85)	0.803
Mental disorders	78	1.20 (0.59 - 2.44)	0.624	0.94 (0.43 - 2.05)	0.882

Vaccine >=3 dose					
		Univariate regression analysis		Multivariable regression analysis	
Potential Risk Factors	N[a]	OR (95% CI)	P-value	aOR (95% CI)	P-value
FH screened by FAMCAT	-	Inf (0.00 – Inf)	0.521	Inf (0.00 - Inf)	0.420
Vaccine status					
0-1 dose	0				
2 doses	0				
≥3 doses	993				
Healthcare utilisation within 2 years	-	0.94 (0.89 - 0.98)	0.005*	0.94 (0.89 - 0.99)	0.026*
COVID-19 associated hospitalisation	71	1.45 (0.79 - 2.68)	0.232	1.24 (0.66 - 2.33)	0.499
Charlson Comorbidity Index	-	1.19 (0.99 - 1.42)	0.059	1.15 (0.94 - 1.41)	0.165
Peripheral vascular disease	8	10.88 (2.51 - 47.22)	0.001*	9.79 (2.10 - 45.56)	0.004*
Hypertension	424	0.72 (0.50 - 1.04)	0.082	0.86 (0.55 - 1.35)	0.519
Type 2 diabetes	194	1.10 (0.71 - 1.73)	0.665	1.25 (0.75 - 2.09)	0.400
Mental disorders	67	1.40 (0.72 - 2.74)	0.323	1.36 (0.67 - 2.77)	0.400

Note: OR: odds ratio, aOR: adjusted odds ratio, 95% CI: 95% confidence interval

*** P-value < 0.0001, ** P-value < 0.001, * P-value < 0.05

a Number of observations: Continuous variables are not presenting an observation number

2.7 Supplementary Table S7. Sensitivity analyses with PCR tested positive patients only

Potential Risk Factors	N[a]	Univariate regression analysis		Multivariable regression analysis	
		OR (95% CI)	P-value	aOR (95% CI)	P-value
FH screened by FAMCAT	-	1.49 (0.00 - Inf)	0.984	435.96 (0.00 – Inf)	0.763
Vaccine status					
0-1 dose	701	ref		ref	
2 doses	992	0.51 (0.37 - 0.70)	<0.001***	0.56 (0.41 - 0.78)	<0.001**
≥3 doses	602	0.45 (0.30 - 0.67)	<0.001**	0.51 (0.33 - 0.77)	0.002*
Healthcare utilisation within 2 years	-	0.95 (0.92 - 0.98)	0.002*	0.95 (0.91 - 0.98)	0.004*
COVID-19 associated hospitalisation	290	1.70 (1.19 - 2.43)	0.004	1.37 (0.94 - 1.98)	0.099
Charlson Comorbidity Index	-	1.10 (1.01 - 1.20)	0.032	1.02 (0.93 - 1.13)	0.675
Peripheral vascular disease	15	2.17 (0.66 - 7.13)	0.203	1.63 (0.48 - 5.51)	0.431
Hypertension	1008	0.94 (0.73 - 1.22)	0.658	1.09 (0.80 - 1.48)	0.572
Type 2 diabetes	518	1.38 (1.04 - 1.85)	0.027	1.40 (1.01 - 1.94)	0.043*
Mental disorders	215	1.18 (0.78 - 1.78)	0.429	1.01 (0.66 - 1.54)	0.980

Note: OR: odds ratio, aOR: adjusted odds ratio, 95% CI: 95% confidence interval

*** P-value < 0.0001, ** P-value < 0.001, * P-value < 0.05

a Number of observations: Continuous variables are not presenting an observation number

2.8 Supplementary Table S8. Sensitivity analyses with cases identification period extended to 22-180 days

Potential Risk Factors	N[a]	Univariate regression analysis		Multivariable regression analysis	
		OR (95% CI)	P-value	aOR (95% CI)	P-value
FH screened by FAMCAT	-	82.11 (0.00 - Inf)	0.615	128.04 (0.00 – Inf)	0.585
Vaccine status					
0-1 dose	1107	ref		ref	
2 doses	1967	0.66 (0.52 - 0.83)	<0.001**	0.68 (0.54 - 0.87)	0.002*
≥3 doses	1242	0.56 (0.42 - 0.74)	<0.001***	0.58 (0.43 - 0.77)	<0.001**
Healthcare utilisation within 2 years	-	0.97 (0.95 - 0.99)	0.011*	0.98 (0.95 - 1.01)	0.114
COVID-19 associated hospitalisation	295	1.36 (0.97 - 1.92)	0.079	1.21 (0.84 - 1.74)	0.301
Charlson Comorbidity Index	-	1.02 (0.93 - 1.11)	0.683	0.96 (0.87 - 1.07)	0.499
Peripheral vascular disease	23	4.55 (1.90 - 10.90)	<0.001**	4.50 (1.81 - 11.16)	0.001*
Hypertension	1933	0.85 (0.70 - 1.03)	0.094	0.94 (0.75 - 1.18)	0.576
Type 2 diabetes	880	1.00 (0.79 - 1.26)	0.975	1.04 (0.79 - 1.36)	0.804
Mental disorders	329	1.03 (0.72 - 1.46)	0.887	0.93 (0.65 - 1.34)	0.691

Note: OR: odds ratio, aOR: adjusted odds ratio, 95% CI: 95% confidence interval

*** P-value < 0.0001, ** P-value < 0.001, * P-value < 0.05

a Number of observations: Continuous variables are not presenting an observation number

2.9 Supplementary Table S9. Sensitivity analyses with matching process without 1:10 limit

Potential Risk Factors	N[a]	Univariate regression analysis		Multivariable regression analysis	
		OR (95% CI)	P-value	aOR (95% CI)	P-value
FH screened by FAMCAT	-	5.08 (0.00 – Inf)	0.896	23.17 (0.00 - Inf)	0.777
Vaccine status					
0-1 dose	3214	ref		ref	
2 doses	6233	0.57 (0.44 - 0.74)	<0.001***	0.63 (0.48 - 0.82)	<0.001**
≥3 doses	3092	0.48 (0.35 - 0.66)	<0.001***	0.54 (0.39 - 0.76)	<0.001**
Healthcare utilisation within 2 years	-	0.95 (0.92 - 0.97)	<0.001***	0.94 (0.91 - 0.97)	<0.001**
COVID-19 associated hospitalisation	537	1.68 (1.24 - 2.27)	<0.001**	1.39 (1.02 - 1.90)	0.039*
Charlson Comorbidity Index	-	1.10 (1.02 - 1.19)	0.011*	1.05 (0.96 - 1.14)	0.295
Peripheral vascular disease	55	3.58 (1.63 - 7.85)	0.001*	3.04 (1.38 - 6.69)	0.006*
Hypertension	5585	0.84 (0.69 - 1.03)	0.094	1.05 (0.82 - 1.33)	0.706
Type 2 diabetes	2626	1.15 (0.91 - 1.45)	0.244	1.17 (0.90 - 1.52)	0.229
Mental disorders	916	1.20 (0.86 - 1.67)	0.283	1.08 (0.77 - 1.52)	0.651

Note: OR: odds ratio, aOR: adjusted odds ratio, 95% CI: 95% confidence interval

*** P-value < 0.0001, ** P-value < 0.001, * P-value < 0.05

a Number of observations: Continuous variables are not presenting an observation number

2.10 Supplementary Table S10. Sensitivity analyses with a threshold of 0.0047 for FAMCAT to screen FH

Potential Risk Factors	N[a]	Univariate regression analysis		Multivariable regression analysis	
		OR (95% CI)	P-value	aOR (95% CI)	P-value
FH screened by FAMCAT	132	0.89 (0.49 - 1.61)	0.687	0.96 (0.53 - 1.75)	0.894
Vaccine status					
0-1 dose	1089	Ref		ref	
2 doses	1421	0.60 (0.47 - 0.79)	<0.001**	0.68 (0.52 - 0.88)	0.004*
≥3 doses	1368	0.49 (0.35 - 0.68)	<0.001***	0.56 (0.40 - 0.79)	<0.001**
Healthcare utilisation within 2 years	-	0.94 (0.92 - 0.97)	<0.001***	0.95 (0.92 - 0.97)	<0.001**
COVID-19 associated hospitalisation	360	1.66 (1.22 - 2.26)	0.001*	1.41 (1.03 - 1.93)	0.035*
Charlson Comorbidity Index	-	1.10 (1.02 - 1.19)	0.018*	1.04 (0.95 - 1.14)	0.367
Peripheral vascular disease	28	3.62 (1.62 - 8.10)	0.002*	2.99 (1.31 - 6.80)	0.009*
Hypertension	1794	0.83 (0.67 - 1.01)	0.062	1.01 (0.80 - 1.29)	0.916
Type 2 diabetes	862	1.13 (0.89 - 1.43)	0.323	1.16 (0.89 - 1.51)	0.276
Mental disorders	340	1.20 (0.86 - 1.68)	0.291	1.09 (0.77 - 1.54)	0.631

Note: OR: odds ratio, aOR: adjusted odds ratio, 95% CI: 95% confidence interval

*** P-value < 0.0001, ** P-value < 0.001, * P-value < 0.05

a Number of observations: Continuous variables are not presenting an observation number

2.11 Supplementary Table S11. Additional analysis stratified by vaccine subtypes

Potential Risk Factors	N[a]	Univariate regression analysis		Multivariable regression analysis	
		OR (95% CI)	P-value	aOR (95% CI)	P-value
1 dose Sinovac-CoronaVac (S)	369	0.51 (0.34 - 0.77)	0.001*	0.55 (0.36 - 0.83)	0.005*
1 dose BNT162b2 (B)	101	0.54 (0.27 - 1.08)	0.082	0.59 (0.29 - 1.19)	0.142
2 doses Sinovac-CoronaVac (SS)	935	0.52 (0.38 - 0.71)	<0.001***	0.60 (0.43 - 0.82)	0.002*
2 doses BNT162b2 (BB)	527	0.40 (0.27 - 0.59)	<0.001***	0.45 (0.30 - 0.68)	<0.001**
3 doses Sinovac-CoronaVac (SSS)	731	0.43 (0.30 - 0.62)	<0.001***	0.50 (0.34 - 0.73)	<0.001**
3 doses BNT162b2 (BBB)	494	0.38 (0.25 - 0.59)	<0.001***	0.46 (0.30 - 0.72)	<0.001**
2 doses Sinovac-CoronaVac followed by 1 dose BNT162b2 (SSB)	156	0.20 (0.09 - 0.41)	<0.001***	0.23 (0.11 - 0.48)	<0.001**
2 doses BNT162b2 followed by 1 dose Sinovac-CoronaVac (BBS)	9	0.33 (0.04 - 2.77)	0.304	0.37 (0.04 - 3.22)	0.371

Note: OR: odds ratio, aOR: adjusted odds ratio, 95% CI: 95% confidence interval

*** P-value < 0.0001, ** P-value < 0.001, * P-value < 0.05

a Number of observations

2.12 Supplementary Table S12. Additional analysis with antiviral treatments included

Potential Risk Factors	N[a]	Univariate regression analysis		Multivariable regression analysis	
		OR (95% CI)	P-value	aOR (95% CI)	P-value
FH screened by FAMCAT	-	0.142 (0 - Inf)	0.878	0.51 (0.00 - Inf)	0.951
Vaccine status					
0-1 dose	1089	Ref		ref	
2 doses	1421	0.60 (0.47 - 0.79)	<0.001**	0.68 (0.52 - 0.88)	0.004*
≥3 doses	1368	0.49 (0.35 - 0.68)	<0.001***	0.56 (0.40 - 0.79)	<0.001**
Healthcare utilisation within 2 years	-	0.94 (0.92 - 0.97)	<0.001***	0.95 (0.92 – 0.97)	<0.001**
COVID-19 associated hospitalisation	360	1.66 (1.22 - 2.26)	0.001*	1.42 (1.03 - 1.97)	0.032*
Charlson Comorbidity Index	-	1.10 (1.02 - 1.19)	0.018*	1.04 (0.95 - 1.14)	0.355
Peripheral vascular disease	28	3.62 (1.62 - 8.10)	0.002*	2.96 (1.30 - 6.75)	0.010*
Hypertension	1794	0.83 (0.67 - 1.01)	0.062	1.01 (0.80 - 1.29)	0.914
Type 2 diabetes	862	1.13 (0.89 - 1.43)	0.323	1.16 (0.89 - 1.50)	0.281
Mental disorders	340	1.20 (0.86 - 1.68)	0.291	1.09 (0.77 - 1.54)	0.627
Antiviral treatments	497	1.09 (0.79 – 1.51)	0.603	0.94 (0.67 – 1.32)	0.720

Note: OR: odds ratio, aOR: adjusted odds ratio, 95% CI: 95% confidence interval

*** P-value < 0.0001, ** P-value < 0.001, * P-value < 0.05

a Number of observations: Continuous variables are not presenting an observation number

2.13 Supplementary Table S13. Post hoc analysis with severity indicators included

Potential Risk Factors	N[a]	Univariate regression analysis		Multivariable regression analysis	
		OR (95% CI)	P-value	aOR (95% CI)	P-value
FH screened by FAMCAT	-	0.142 (0 - Inf)	0.878	0.80 (0 - Inf)	0.984
Vaccine status					
0-1 dose	1089	Ref		ref	
2 doses	1421	0.60 (0.47 - 0.79)	<0.001**	0.67 (0.52 - 0.88)	0.004*
≥3 doses	1368	0.49 (0.35 - 0.68)	<0.001***	0.57 (0.40 - 0.79)	<0.001**
Healthcare utilisation within 2 years	-	0.94 (0.92 - 0.97)	<0.001***	0.95 (0.92 – 0.97)	<0.001**
COVID-19 associated hospitalisation	360	1.66 (1.22 - 2.26)	0.001*	1.90 (1.06 – 3.40)	0.030*
Charlson Comorbidity Index	-	1.10 (1.02 - 1.19)	0.018*	1.04 (0.95 - 1.14)	0.408
Peripheral vascular disease	28	3.62 (1.62 - 8.10)	0.002*	2.71 (1.17 - 6.25)	0.020*
Hypertension	1794	0.83 (0.67 - 1.01)	0.062	1.02 (0.80 - 1.29)	0.904
Type 2 diabetes	862	1.13 (0.89 - 1.43)	0.323	1.16 (0.89 - 1.51)	0.261
Mental disorders	340	1.20 (0.86 - 1.68)	0.291	1.09 (0.77 - 1.55)	0.616
Cardiac injury markers	199	1.79 (1.22 – 2.62)	0.003*	1.37 (0.84 - 2.23)	0.205
COVID-19 associated pneumonia	298	1.37 (0.97 – 1.93)	0.073	0.56 (0.29 - 1.06)	0.076
ICU admission	5	1.40 (0.14 – 13.75)	0.772	1.07 (0.10 - 11.99)	0.954

Note: OR: odds ratio, aOR: adjusted odds ratio, 95% CI: 95% confidence interval

*** P-value < 0.0001, ** P-value < 0.001, * P-value < 0.05

a Number of observations: Continuous variables are not presenting an observation number

3. Supplementary References

1. McGowan MP, Hosseini Dehkordi SH, Moriarty PM, Duell PB. Diagnosis and treatment of heterozygous familial hypercholesterolemia. Journal of the American Heart Association. 2019;8(24):e013225.
2. Akyea RK, Qureshi N, Kai J, Lusignan Sd, Sherlock J, McGee C, et al. Evaluating a clinical tool (FAMCAT) for identifying familial hypercholesterolaemia in primary care: a retrospective cohort study. 2020;4(5):bjgpopen20X101114.
3. Weng S, Kai J, Akyea R, Qureshi NJTLPH. Detection of familial hypercholesterolaemia: external validation of the FAMCAT clinical case-finding algorithm to identify patients in primary care. 2019;4(5):e256-e64.
4. Qureshi N, Akyea RK, Dutton B, Leonardi-Bee J, Humphries SE, Weng S, et al. Comparing the performance of the novel FAMCAT algorithms and established case-finding criteria for familial hypercholesterolaemia in primary care. 2021;8(2):e001752.
5. Weng SF, Kai J, Andrew Neil H, Humphries SE, Qureshi N. Improving identification of familial hypercholesterolaemia in primary care: Derivation and validation of the familial hypercholesterolaemia case ascertainment tool (FAMCAT). Atherosclerosis. 2015;238(2):336-43.
